# The role of neuronal network synchronization as a potential biomarker for bipolar disorder

**DOI:** 10.1192/j.eurpsy.2023.1476

**Published:** 2023-07-19

**Authors:** M. Mnif, N. Smaoui, L. Triki, D. Jardak, I. Gassara, R. Feki, S. Omri, M. Maalej, N. Charfi, J. Ben Thabeut, L. Zouari, K. Masmoudi, M. Maalej

**Affiliations:** 1 Psychiatry C department, Hedi Chaker Hospital; 2 Functional exploration department, Hbib Borguiba Hospital, Sfax, Tunisia

## Abstract

**Introduction:**

Despite the potential for EEG abnormalities to provide insight into the neurophysiology of disease processes, studies that measure EEG power and coherence in bipolar disorder (BD) are rare.

**Objectives:**

We investigated whether the resting electroencephalogram (EEG) in patients with BD showed altered synchronization

**Methods:**

This was a cross-sectional, descriptive, and analytical case-control study, conducted with patients followed for BD in the psychiatry department "C" at the Hedi Chaker hospital in Sfax compared to healthy controls. Patients were assessed by the Hamilton Depression Scale (HDRS-17), and the Young Mania Scale (YMRS). EEG was also recorded at the service of the functional exploration at the Habib Bourguiba hospital in Sfax. Functional connectivity between pairs of EEG channels was measured for 4 frequency bands delta [0.5 – 3.5 Hz], theta [4 – 7.5 Hz], alpha [8 – 12.5 Hz], and beta [13 – 30 Hz]. Statistical analyses were carried out.

**Results:**

Thirty subjects including 15 patients with BD and 15 age- and sex-matched controls were included. The mean age of bipolar and control was 36.07 ± 10.50 and 47.93 ± 15.61 years, respectively. The mean scores on the HDRS-17 and YMRS were 2.73±2.08, and 1.67±3.53 respectively.

Bipolar patients showed a decrease of connectivity in **the delta band**, and the decreases were greatest between the left frontal lobe and the right frontal, parietal and temporal lobes on the one hand and between the left temporal and right parietal lobes on the other hand. For **the theta band**, there was poor connectivity between the left frontal lobe and the right frontal and temporal lobes on the one hand and between the right central area and the left parietal, temporal and occipital lobes.

**Conclusions:**

Bipolar patients had poorer intra and interhemispheric connectivity, which may be a key feature of BD.

**Disclosure of Interest:**

None Declared

